# Prevalence and risk factors of severe postpartum hemorrhage: a retrospective cohort study

**DOI:** 10.1186/s12884-021-03818-1

**Published:** 2021-04-26

**Authors:** Chen-ning Liu, Fu-bing Yu, Yun-zhe Xu, Jin-sheng Li, Zhi-hong Guan, Man-na Sun, Chen-an Liu, Fang He, Dun-jin Chen

**Affiliations:** 1 Department of Obstetrics and Gynecology, The Third Affiliated Hospital of Guangzhou Medical University, Guangzhou, 510150 China; 2 Dongguan Maternal and Child Health Care Hospital, Dongguan, China; 3 Shanxi Medical University, Taiyuan, China; 4 Key Laboratory for Major Obstetric Diseases of Guangdong Province, Guangzhou, 510150 China; 5 Guangzhou Medical Centre for Critical Pregnant Women, Guangzhou, China; 6 Quality Control Center of Obstetrics of Guangdong Province, Guangzhou, China

**Keywords:** Postpartum hemorrhage, Causes, Risk factors

## Abstract

**Background:**

Although maternal deaths are rare in developed regions, the morbidity associated with severe postpartum hemorrhage (SPPH) remains a major problem. To determine the prevalence and risk factors of SPPH, we analyzed data of women who gave birth in Guangzhou Medical Centre for Critical Pregnant Women, which received a large quantity of critically ill obstetric patients who were transferred from other hospitals in Southern China.

**Methods:**

In this study, we conducted a retrospective case-control study to determine the prevalence and risk factors for SPPH among a cohort of women who gave birth after 28 weeks of gestation between January 2015 and August 2019. SPPH was defined as an estimated blood loss ≥1000 mL and total blood transfusion≥4 units. Logistic regression analysis was used to identify independent risk factors for SPPH.

**Results:**

SPPH was observed in 532 mothers (1.56%) among the total population of 34,178 mothers. Placenta-related problems (55.83%) were the major identified causes of SPPH, while uterine atony without associated retention of placental tissues accounted for 38.91%. The risk factors for SPPH were maternal age < 18 years (adjusted OR [aOR] = 11.52, 95% CI: 1.51–87.62), previous cesarean section (aOR = 2.57, 95% CI: 1.90–3.47), history of postpartum hemorrhage (aOR = 4.94, 95% CI: 2.63–9.29), conception through in vitro fertilization (aOR = 1.78, 95% CI: 1.31–2.43), pre-delivery anemia (aOR = 2.37, 95% CI: 1.88–3.00), stillbirth (aOR = 2.61, 95% CI: 1.02–6.69), prolonged labor (aOR = 5.24, 95% CI: 3.10–8.86), placenta previa (aOR = 9.75, 95% CI: 7.45–12.75), placenta abruption (aOR = 3.85, 95% CI: 1.91–7.76), placenta accrete spectrum (aOR = 8.00, 95% CI: 6.20–10.33), and macrosomia (aOR = 2.30, 95% CI: 1.38–3.83).

**Conclusion:**

Maternal age < 18 years, previous cesarean section, history of PPH, conception through IVF, pre-delivery anemia, stillbirth, prolonged labor, placenta previa, placental abruption, PAS, and macrosomia were risk factors for SPPH. Extra vigilance during the antenatal and peripartum periods is needed to identify women who have risk factors and enable early intervention to prevent SPPH.

## Background

Severe postpartum hemorrhage (SPPH) is the leading cause of maternal deaths and severe maternal morbidities, accounting for 27.1% of maternal deaths worldwide, ranging from 8% in developed areas to 32% in Northern Africa [1]. The incidence of postpartum hemorrhage (PPH) ranges from 3 to 8%, and the increasing rate is a growing public concern [2–4]. Severe complications such as hemorrhagic shock, acute respiratory distress syndrome, disseminated intravascular coagulation, acute renal failure, loss of fertility, pituitary necrosis (Sheehan syndrome), and even maternal death may be caused by delayed recognition or improper clinical procedures.

Most deaths resulting from SPPH occur during the first 24 h after birth. It is generally assumed that most SPPH-associated deaths could be avoided by the prevention and timely treatment of SPPH. The hemorrhage transition from the compensated to the decompensated stage is rapid and easily overlooked [5]. Hence, prediction, early recognition and intervention are crucial to reduce the likelihood of SPPH or improve the clinical outcomes of SPPH [6]. The definition of SPPH varies between different guidelines, for example, blood loss over 1000 mL, 1500 mL, 2000 mL, 30–40% of total blood volume, or over 4 units of transfusion [6–8]. Commonly used definitions of PPH are based on estimated blood loss within 24 h of childbirth [9, 10]. However, the severity of PPH depends not only on the quantity of bleeding, but also on the bleeding rate, physical conditions of the mothers, physiological response to bleeding and medical conditions. An inaccurately estimated blood loss may result in the SPPH being not fully diagnosed.

Although SPPH may develop unexpectedly, many studies have attempted to alert doctors by identifying specific high-risk factors for PPH. Some experts suggested that risk factors for PPH should be classified as high, medium, or low risk, in order to alert obstetricians [11]. The risk factors of PPH vary in different studies and guidelines [4, 12–14]. Risk-assessment tools to predict women at risk for PPH, such as CMCQQ (California Maternal Quality Care Collaborative), AWHONN (Association of Women’s Health, Obstetric and Neonatal Nurses) and NYBOH (New York Safety Bundle for Obstetric Hemorrhage), have been widely used in obstetrics [15]. However, data regarding the validity of these safety bundles are limited. Kawakita et al. found that these tools had a moderate predictive power to identify women at a high risk for SPPH after cesarean delivery [15].

To date, there is still a lack of a reliable assessment tool to accurately screen high-risk women with SPPH. In this study, we analyzed a retrospective cohort study of women who gave birth in Guangzhou Medical Centre for Critical Pregnant Women, which received a large quantity of critically ill obstetric patients who were transferred from other hospitals in Southern China. We used the criteria of severe maternal morbidities [16], which was defined by an estimated blood loss and total use of ≥4 units of transfused blood, to determine the prevalence and risk factors of SPPH.

## Methods

### Study population

Ethical approval (approval number: [2020] 055) was obtained for this retrospective analysis, and the need for informed consent was waived due to the retrospective nature of this study. This study comprised of an evaluation of the institutional perinatology database of all women giving birth after 28 weeks of gestation in the Third Affiliated Hospital of Guangzhou Medical University (Guangzhou Medical Centre for Critical Pregnant Women) from January 2015 to August 2019 (34,178 mothers). Administrative permission from the Third Affiliated Hospital of Guangzhou Medical University was acquired by our team to access the institutional perinatology database used in our research. Management of PPH in our center (since 2015) follows the guidelines of the Management and Prevention of Postpartum Hemorrhage published by the Gynecology and Obstetrics of Chinese Medical Association [17]. The database used in this study was derived from medical records and contained information on maternal health before and during pregnancy, detailed information about delivery and complications occurring intrapartum or postpartum and information about the newborns. Volume of PPH, transfusion information, modes of delivery, and various candidate risk factors were registered, thereby facilitating case ascertainment. Other diseases were identified by the presence of diagnostic codes from the International Classification of Diseases, 10th Revision, Clinical Modification (ICD-10-CM).

From this population, we conducted a retrospective case-control study. The outcome was SPPH, which was defined as blood loss exceeding 1000 mL within 24 h of childbirth, and women received either≥4 units of RBCs or a multicomponent blood transfusion. Blood loss was measured by combining visual estimations, the gravimetric method, the estimated blood loss volume, shock index, and hemoglobin levels by attending clinical caregivers, namely physicians, midwives, and nurses. Estimated blood loss was recorded by a resident physician present at delivery. The deadline for an eventual blood transfusion was the time of discharge. A multicomponent blood transfusion was defined as a blood transfusion consisting of a combination of RBCs and fresh frozen plasma and/or platelet concentrates.

Based on a review of the relevant literature and clinical plausibility, we formulated a set of 27 candidate risk factors for maternal characteristics and comorbidities that potentially contributed to increased risk of SPPH. We restricted our analysis of potential risk factors that would likely be identifiable from the antepartum period until the delivery was completed and were not complications developing from the delivery. We defined the presence of each condition as having one or more corresponding codes during this period. In the study population of 34,178 mothers, there were 844 women excluded from the risk factor analysis due to incomplete information.

The explanatory variables (explained in Appendix) included demographic, pre-gestational medical and obstetric-related factors. The demographic characteristics were as follows: age of delivery, history of cesarean delivery, parity, body mass index (BMI) before pregnancy and history of PPH. The pre-gestational medical variables were defined as medical diseases prior to pregnancy, and diseases including chronic hypertension, pre-gestational diabetes mellitus (PGDM) and cardiac diseases. Pregnancy-related variables were identified directly in ticked boxes, such as in vitro fertilization (IVF), regular or irregular prenatal examination, singleton or twin pregnancy, hypertensive disorders of pregnancy, HELLP syndrome, gestational diabetes mellitus (GDM), fibroids, anemia, thrombocytopenia, blood coagulation disorder, stillbirth, placenta previa, placenta abruption, placenta accrete spectrum (PAS) and macrosomia. Labor-related obstetric variables included induction of labor, prolonged labor, precipitate labor, and mode of delivery.

### Statistical analysis

The prevalence and causes of SPPH were expressed as number (n) and percentage (% or ‰). Cross-tabulations were used to show the proportions of demographic, medical and obstetric factors in women with SPPH and controls. Univariate logistic analysis was performed to assess candidate variables as risk factors for SPPH, and the associations between potential risk factors and SPPH were quantified by the odds ratio (OR) and 95% confidence interval (CI). All explanatory variables with a significance level of *P* < 0.05 in univariate analysis were included in the multivariate logistic analysis. In consideration of previous reports and clinical plausibility, we especially included twins, thrombocytopenia, blood coagulation disorder, precipitate labor and macrosomia in multivariate analysis, even if they had a *P* ≥ 0.05 on univariate analysis. The data were analyzed using SPSS version 24. Statistical significance was set at *P* < 0.05.

## Results

SPPH was observed in 532 mothers (1.56%) among the total population of 34,178 mothers. Placenta-related causes (55.83%) were the primary identified causes of SPPH, while uterine atony without associated retention of placental tissues accounted for 38.91% of the cases. Trauma and coagulopathy could be identified in 2.82 and 1.13% of the SPPH cases, respectively.

### Risk factors of SPPH

A total of 844 mothers were excluded, and 33,334 women were included in this analysis. The study population comprised a total of 506 cases of SPPH and 32,828 controls without SPPH. The distribution of potential risk factors is presented in Table 1. In the univariate analysis, SPPH was more likely among mothers with the following characteristics: ≥35y, multiparous, history of PPH, previous cesarean delivery, conception through IVF, irregular prenatal examination, GDM, anemia, stillbirth, prolonged labor, cesarean section, placental previa, placental abruption and PAS.

**Table 1:** Clinical profile of women with severe postpartum hemorrhage versus controls

	SPPH(*n* = 506)	Controls (*n* = 32,828)	OR	95%CI	*P*
Maternal age (y)					
< 18	1 (0.20%)	20 (0.06%)	3.99	0.53–29.84	0.636
18–34.9	318 (62.85%)	25,389 (77.34%)	Ref.		
35–39.9	149 (29.45%)	5883 (17.92%)	2.02	1.66–2.46	< 0.001
≥ 40	38 (7.51%)	1536 (4.68%)	1.98	1.41–2.78	< 0.001
Parity					
0	138 (27.27%)	17,913 (54.57%)	Ref.		
1–2	355 (70.16%)	14,698 (44.77%)	3.14	2.57–3.82	< 0.001
≥ 3	13 (2.57%)	217 (0.66%)	7.78	4.34–13.95	< 0.001
Previous cesarean delivery^a^	302 (81.84%)	6323 (42.34%)	6.14	4.70–8.01	< 0.001
BMI (kg/m^2^)					
< 18.5	41 (8.10%)	3239 (9.87%)	0.83	0.60–1.14	0.250
18.5–24.9	354 (69.96%)	23,107 (70.39%)	Ref.		
25–29.9	91 (17.98%)	5531 (16.85%)	1.07	0.85–1.36	0.547
≥ 30	20 (3.95%)	951 (2.90%)	1.37	0.87–2.16	0.172
History of PPH^b^	20 (5.42%)	161 (1.08%)	5.26	3.27–8.47	< 0.001
Chronic hypertension	0 (0.00%)	79 (0.24%)	–	–	0.519
PGDM	0 (0.00%)	248 (0.76%)	–	–	0.089
Cardiac disease	8 (1.58%)	354 (1.08%)	1.47	0.73–2.99	0.282
IVF	84 (16.60%)	4283 (13.05%)	1.33	1.05–1.68	0.019
Irregular prenatal examination	282 (55.73%)	15,524 (47.29%)	1.40	1.18–1.68	< 0.001
Twins	34 (6.72%)	2186 (6.66%)	1.01	0.71–1.43	0.957
Hypertensive disorders of pregnancy					
Gestational hypertension	15 (2.96%)	706 (2.15%)	1.40	0.83–2.36	0.203
Preeclampsia	26 (5.14%)	1422 (4.33%)	1.21	0.81–1.80	0.356
Eclampsia	0 (0.00%)	18 (0.05%)	–	–	0.999
HELLP syndrome	2 (0.40%)	68 (0.21%)	1.91	0.47–7.82	0.669
GDM	105 (20.75%)	5369 (16.35%)	1.34	1.08–1.66	0.008
Fibroids	5 (0.99%)	172 (0.52%)	1.90	0.78–4.63	0.264
Anemia	158 (31.23%)	2954 (9.00%)	4.59	3.79–5.56	< 0.001
Thrombocytopenia	6 (1.13%)	317 (0.97%)	1.23	0.55–2.77	0.785
Blood coagulation disorder	2 (0.40%)	43 (0.13%)	3.03	0.73–12.52	0.319
Stillbirth	7 (1.38%)	132 (0.40%)	3.48	1.62–7.47	0.002
Induction of labor	22 (4.35%)	3045 (9.28%)	0.45	0.29–0.68	< 0.001
Prolonged labor	18 (3.56%)	583 (1.78%)	2.04	1.27–3.29	0.003
Precipitate labor	1 (0.20%)	219 (0.67%)	0.30	0.04–2.11	0.309
Mode of delivery					
Vaginal delivery	370 (73.12%)	27,780 (84.62%)	Ref.		
Cesarean section	136 (26.88%)	5408 (16.47%)	2.02	1.66–2.47	< 0.001
Placenta previa	311 (61.46%)	1392 (4.24%)	36.02	29.88–43.42	< 0.001
Placental abruption	11 (2.17%)	337 (1.03%)	2.14	1.17–3.93	0.014
PAS	283 (55.93%)	1076 (3.28%)	37.45	31.10–45.09	< 0.001
Macrosomia	41 (8.10%)	2553 (7.78%)	1.05	0.76–1.44	0.79

Data presented as n (%), odds ratio (OR) and 95% confidence intervals (CI)

Previous cesarean delivery^a^: nulliparous women were removed from the denominator, 302/369 (81.84%) in SPPH, and 6323/14934 (42.34%) in controls

History of PPH^b^: nulliparous women were removed from the denominator, 20/369 (5.42%) in SPPH, and 161/14934 (1.08%) in controls

*SPPH* severe postpartum hemorrhage, *BMI* body mass index, *PGDM* pre-gestational diabetes mellitus, *IVF* in vitro fertilization, *HELLP* syndrome, hemolysis elevated liver enzymes, low platelet count, *GDM* gestational diabetes mellitus, *PAS* placenta accrete spectrum

Risk factors independently associated with SPPH are showed in Table 2. The risk of SPPH increased significantly in those aged < 18 years (adjusted ratio [aOR] = 11.52). The risk of mothers with a history of PPH and previous cesarean section elevated, and the aOR was 4.94 and 2.57, respectively. Placental factors were significantly associated with SPPH; placental previa had the highest adjusted odds ratio of SPPH (9.75), followed by PAS (8.00) and placental abruption (3.85). Other obstetric risk factors included prolonged labor (aOR = 5.24), stillbirth (aOR = 2.61), anemia (aOR = 2.37), macrosomia (aOR = 2.30), and IVF (aOR = 1.78). In addition, cesarean section was a protective factor in this study, with an aOR of 0.58.

**Table 2:** Multivariable logistic model for SPPH

Independent risk factors	Adjusted OR	95% CI	*P*
Maternal age < 18y	11.52	1.51–87.62	0.018
Previous cesarean section	2.57	1.90–3.47	< 0.001
History of PPH	4.94	2.63–9.29	< 0.001
IVF	1.78	1.31–2.43	< 0.001
Anemia	2.37	1.88–3.00	< 0.001
Stillbirth	2.61	1.02–6.69	0.045
Prolonged labor	5.24	3.10–8.86	< 0.001
Cesarean section	0.58	0.46–0.74	< 0.001
Placenta previa	9.75	7.45–12.75	< 0.001
Placental abruption	3.85	1.91–7.76	< 0.001
PAS	8.00	6.20–10.33	< 0.001
Macrosomia	2.30	1.38–3.83	0.001

*IVF* in vitro fertilization, *PAS* placenta accrete spectrum

## Discussion

### Prevalence of SPPH

In our study, the prevalence of SPPH was 1.56%, which is in accordance with the known prevalence of 0.3–5.1% [4, 13, 14, 18]. The rates of SPPH vary according to time and geographical region. SPPH is a major threat to maternal health. It has been reported that compared to women without SPPH, those with SPPH had 116 times, 87 times, and 5.3 times the risk of hysterectomy, acute renal failure, and sepsis, respectively, along with an increased frequency of ICU admission, reflecting the severity and potential lethality of this complication [14].

The definition of SPPH varies according to different guidelines. The most commonly accepted definition of SPPH is based on the amount of blood loss after birth. The WHO recommends visual estimation of blood loss as the standard for blood loss measurement; yet, visual estimates underestimate blood loss volumes by 33–50% when compared with spectrophotometry [19, 20]. Moreover, the severity of PPH depends not only on the quantity of bleeding, but also on the bleeding rate, physical conditions of the mother, physiological response to bleeding and medical conditions. An inaccurately estimated blood loss may result in the SPPH being not fully diagnosed. We used a blood loss volume ≥ 1000 mL and blood transfusions≥4 units as the cutoffs for SPPH in order to reduce bias and make our results more reliable.

Common causes of PPH include uterine atony, placenta-related problems, trauma and failure of the blood coagulation system. In our study, uterine atony without other associated causes was identified in only 38.91% of mothers, which was much lower than the reported prevalence of 70–80% [4, 21, 22]. Meanwhile, abnormal placentation was responsible for the majority (55.83%) of SPPH, which was much higher than the previously reported prevalence of 10% [4, 22]. This might be explained by our classification rules and the special patient population in our hospital. In our study, women who had atony due to retained placental tissues were categorized into the group of placental-related causes. In addition, our hospital, Guangzhou Medical Centre for Critical Pregnant Women, receives a large number of critically ill obstetric patients transferred from other hospitals in Southern China, including a high proportion of cases with PAS and dangerous placenta previa. As previous studies reported, the prevalence of PAS and placenta previa were 0.1–11 per 1000 deliveries and 5.5 per 1000 deliveries, respectively [23]. In our study, there were 306 mothers (57.52%) with PAS and 332 cases (62.41%) with placenta previa in the SPPH group, which were much higher than the proportions in other hospitals. Our findings suggest that placental-related cause might be a more prominent cause of SPPH than previously reported. Trauma accounted for only 2.82%, which was less than the 20% proportion typically reported [14]. This could be partly due to proper obstetric care though labor and vaginal delivery procedures. Coagulopathy prevalence was relatively in accordance with a known prevalence of 1% [14].

### Risk factors for SPPH

The risk factors for SPPH were maternal age < 18 years, a previous cesarean section, history of PPH, conception through IVF, pre-delivery anemia, stillbirth, prolonged labor, placenta previa, placental abruption, PAS and macrosomia. Previous cesarean section, pre-delivery anemia, stillbirth, prolonged labor and macrosomia were associated with SPPH, consistent with the results of previous reports [4, 12–14, 24–26]. With antenatal anemia affecting up to 25% of pregnant women, initiatives may be necessary to promote anemia correction [27]. Several professional societies, including the Royal College of Obstetricians and Gynecologists (UK) and the French College of Gynecologists and Obstetricians, have published PPH guidelines recommending that a hemoglobin level above 8 g/dL be set as the therapeutic goal [28]. Compared with expectant management, the active management of the third stage of labor is associated with a substantial reduction in the occurrence of PPH [29]. Controlled cord traction (CCT), prophylactic oxytocin, and prophylactic uterine massage are effective methods in the third stage of labor. If the placenta is retained and bleeding occurs, manual removal of the placenta should be expedited [30]. Macrosomia is known to overdistend the uterus which is associated with uterine atony. Macrosomia is an increasingly common lifestyle problem needing public health intervention, and is associated with high BMIs, geriatric pregnancies and diabetes mellitus [31, 32].

Previous studies have reported that advanced maternal age was associated with PPH, but consensus is still lacking on this subject. Kramer et al. stated that maternal age ≥ 35 years (aOR, 1.5; 95% CI, 1.5–1.6) increased the risk of SPPH [33]. Sheen et al. suggested that women with advanced maternal age (≥45 years) were at highest risk for a broad range of adverse outcomes, including PPH, during delivery hospitalizations [34]. However, a meta-analysis indicated that no relationship was found between maternal age ≥ 35 years and PPH (aOR, 1.02; 95% CI, 0.99–1.04) [35]. Moreover, a study in Hong Kong found that advancing age has a protective effect against PPH [36]. In our study, the results showed that older maternal age (≥35y) increased the incidence of SPPH in univariate analysis, but maternal age < 18y was actually associated with increased SPPH. Nevertheless, there was only one woman whose maternal age was < 18 years among the patients with SPPH (0.20%) and 0.06% among controls; therefore, the adjusted ratio had a wide confidence interval. Given the insufficient sample size, our data present cannot conclude that a maternal age < 18y increases the risk of SPPH. Women with a history of PPH had 4.94-fold increased chance of SPPH. Likewise, a study from Australia reported a recurrence rate of 28% from medical audits [37]. A study from Sweden reported that the recurrence of PPH might be explained by environmental and genetic factors [38]. The risk of SPPH elevated in women who conceived through IVF, which was in accordance with previous studies. Zhu et al. reported that placental adherence occurred more frequently in a group after assisted reproductive technology [39].

Our study showed that cesarean section was associated with SPPH, together with an increased incidence of SPPH in the univariate analysis. However, the risk of SPPH decreased by 43% in women who underwent cesarean delivery in the multivariate model. The protective effect of cesarean section was contrary to the results of most previous studies. However, a few studies have reported protective effects of cesarean sections against PPH when compared with vaginal births [40]. Our study showed that the risk of SPPH significantly elevated in women with placenta previa, placental abruption, and PAS, which was consistent with previous studies [4, 12–14, 24–26]. Placenta-related factors contributed significantly to severe forms of PPH, such as PPH with blood transfusion and PPH with hysterectomy.

### Strengths and limitations

Our study has several strengths, as well as some limitations. First, a large cohort of women from the Guangzhou Medical Centre for Critical Pregnant Women ensured a representative sample of critical obstetric patients with complete data from Southern China. Second, we diagnosed SPPH by combining blood loss volumes with blood transfusion to minimize bias; thus, we analyzed the causes and risk factors of SPPH comprehensively and objectively.

The limitations of our study are as follows. The diagnoses in this study were based on ICD codes from insurance claims data, and the main limitation is the validity of the diagnoses in this database. Fibroids and anemia could not be subdivided into different levels. In addition, we lack data on instrumental/spontaneous vaginal deliveries and emergency/elective cesarean sections. Although operation coding existed for forceps, vacuum, and assisted breech interventions, as well as emergency or elective cesarean sections, the operations sometimes were coded by other broader surgery codes and thus failed to identify a substantial proportion of different modes of delivery; this prevented us from studying the contribution of delivery mode to the occurrence of SPPH. Moreover, we did not analyze the impact of smoking or drinking on SPPH, since it was rare in our population.

## Conclusions

Maternal age < 18 years, previous cesarean section, history of PPH, conception through IVF, pre-delivery anemia, stillbirth, prolonged labor, placenta previa, placental abruption, PAS, and macrosomia were risk factors for SPPH. These risk factors may be useful for screen women with a high risk of SPPH. Extra vigilance during the antenatal and peripartum periods is needed to identify women who have risk factors and enable early intervention to prevent SPPH. It is important to remember that we have to prepare for all mothers giving birth, as some get SPPH without any known risk factors.

## Data Availability

The datasets used and/or analyzed during the current study are available from the corresponding authors on reasonable request.

## Appendix

**Table 3:** Part of variables investigated in analysis

Variable	Explanation
Maternal age (y)	age of delivery; categorized into four groups: < 18, 18–34.9, 35–39.9 and ≥ 40 years.
Parity	grouped into no previous deliveries (0), 1–2 previous deliveries, and ≥ 3 previous deliveries.
BMI (kg/m^2^)	low weight < 18.5; normal: 18.5–24.9; overweight: 25–29.9; obesity≥30.
Hypertensive disorders of pregnancy	categorized into gestational hypertension, preeclampsia and eclampsia; gestational hypertension was defined as only if she did not have codes for pre-existing hypertension or preeclampsia or eclampsia.
Anemia	hemoglobin< 9 g/dL before delivery.
Thrombocytopenia	platelet count< 100*10^9^/L.
Prolonged labor	prolonged first stage of labor was determined as deviation of cervical dilatation from the normal rate of 1 cm/hour in the active phase or slow progress of the descent of the presenting part through birth canal; prolonged second stage of labor was> 1 h from complete cervical dilation to delivery if multiparous and > 2 h between complete cervical dilation and delivery if nulliparous.
Precipitate labor	3 h or less from the onset of regular contractions to birth.
Macrosomia	substituted by a birthweight of ≥4 kg.

## Abbreviations

PPH: Postpartum hemorrhage
SPPH: Severe postpartum hemorrhage
PAS: Placenta accrete spectrum
GDM: Gestational diabetes mellitus
PGDM: Pre-gestational diabetes mellitus
BMI: Body mass index
HELLP: Hemolysis elevated liver enzymes, low platelet count
IVF: In vitro fertilization
OR: Odds ratio
aOR: Adjusted odds ratio
CI: Confidence interval
